# Ellagic Acid Derivatives from *Rubus ulmifolius* Inhibit *Staphylococcus aureus* Biofilm Formation and Improve Response to Antibiotics

**DOI:** 10.1371/journal.pone.0028737

**Published:** 2012-01-05

**Authors:** Cassandra L. Quave, Miriam Estévez-Carmona, Cesar M. Compadre, Gerren Hobby, Howard Hendrickson, Karen E. Beenken, Mark S. Smeltzer

**Affiliations:** 1 Department of Microbiology and Immunology, University of Arkansas for Medical Sciences, Little Rock, Arkansas, United States of America; 2 Department of Pharmaceutical Sciences, University of Arkansas for Medical Sciences, Little Rock, Arkansas, United States of America; 3 Department of Orthopaedic Surgery and Center for Orthopaedic Research, University of Arkansas for Medical Sciences, Little Rock, Arkansas, United States of America; 4 Pharmacy Department, National School of Biological Sciences, National Polytechnic Institute, Mexico City, Mexico; National Institutes of Health, United States of America

## Abstract

**Background:**

Biofilms contribute to the pathogenesis of many forms of *Staphylococcus aureus* infection. Treatment of these infections is complicated by intrinsic resistance to conventional antibiotics, thus creating an urgent need for strategies that can be used for the prevention and treatment of biofilm-associated infections.

**Methodology/Principal Findings:**

This study demonstrates that a botanical natural product composition (220D-F2) rich in ellagic acid and its derivatives can limit *S. aureus* biofilm formation to a degree that can be correlated with increased antibiotic susceptibility. The source of this composition is *Rubus ulmifolius* Schott. (Rosaceae), a plant used in complementary and alternative medicine in southern Italy for the treatment of skin and soft tissue infections. All *S. aureus* clonal lineages tested exhibited a reduced capacity to form a biofilm at 220D-F2 concentrations ranging from 50–200 µg/mL, which were well below the concentrations required to limit bacterial growth (530–1040 µg/mL). This limitation was therapeutically relevant in that inclusion of 220D-F2 resulted in enhanced susceptibility to the functionally-distinct antibiotics daptomycin, clindamycin and oxacillin. Testing with kidney and liver cell lines also demonstrated a lack of host cell cytotoxicity at concentrations of 220D-F2 required to achieve these effects.

**Conclusions/Significance:**

These results demonstrate that extract 220D-F2 from the root of *Rubus ulmifolius* can be used to inhibit *S. aureus* biofilm formation to a degree that can be correlated with increased antibiotic susceptibility without toxic effects on normal mammalian cells. Hence, 220D-F2 is a strong candidate for development as a botanical drug for use in the prevention and treatment of *S. aureus* biofilm-associated infections.

## Introduction

*Staphylococcus aureus* is arguably the most problematic of all bacterial pathogens owing in large part to the persistent emergence of antibiotic resistant strains. This is evident in the recent appearance of methicillin-resistant strains even among isolates causing community-acquired infections [1], [2], [3]. While this has created an urgent need for new antimicrobial agents, many *S. aureus* infections are recalcitrant to antimicrobials even in the absence of issues related to acquired-antibiotic resistance. A primary contributing factor to this recalcitrance is the formation of a biofilm. Indeed, the National Institutes of Health estimates that 80% of all bacterial infections are biofilm related [4]. In addition to their impact on antimicrobial therapy, bacteria within biofilms reach a much higher density (10^11^ CFU/mL) than their planktonic counterparts (10^8^ CFU/mL) [5], thus increasing the opportunity for gene transfer and the emergence of strains with new resistance and/or virulence profiles. The presence of a foreign body decreases the minimal infecting dose of *S. aureus* >100,000-fold [6], thus significantly increasing the chances of a biofilm-associated infection by comparison to individuals without such devices. In most cases, treatment of such biofilm-associated infections requires both aggressive antimicrobial treatment and surgical debridement to remove all infected tissues and/or indwelling medical devices [7], [8].

The paradigm of biofilm resistance presents a major hurdle for the treatment of many types of infectious disease, including dental caries, mastitis, otitis media, endocarditis, chronic wounds, and osteomyelitis [5]. The economic burden of these infections is tremendous with biofilm-associated infections lead to longer hospital stays, recurrent infection, and increased fatalities in the most recalcitrant cases. In total, up to 17 million new biofilm infections occur each year in the US, resulting in up to 550,000 fatalities annually [9], [10]. These statistics emphasize the fact that, while there is indeed a pressing need for new antibiotics, there is an equally urgent need to develop agents that could be used to limit biofilm formation to a therapeutically relevant degree. Such agents could be used to prevent colonization or as adjunct therapy to enhance the therapeutic efficacy of conventional antibiotics [10], [11].

Taking an ethnobotanical approach to drug discovery [12] offers considerable promise in many clinical contexts including infectious disease. Indeed, studies on botanical complementary and alternative (CAM) therapies have led to the discovery of novel ant-virulence agents. For instance, proanthocyanidins from *Vaccinium macrocarpon* (cranberry) disrupt adhesion of P-fimbriated *E. coli* to uroepithelial cells [13], [14], accounting for efficacy in preventing recurrent urinary tract infections [15]. Garlic extract (*Allium sativum*) attenuates virulence by inhibiting hyphae formation in *Candida albicans* [16] and by blocking quorum sensing in *Pseudomonas aeruginosa* [17], [18], [19]. In *S. aureus*, inhibitors of quorum sensing, biofilm formation, and the NorA efflux pump have all been isolated from botanical sources [20], [21], [22], [23], [24], [25], [26], [27], [28], [29]. Natural products offer a distinct advantage over their synthetic counterparts due to their rich structural diversity, chirality, and extensive functional group chemistry [24]. Thus, plants are a likely source of the next generation of anti-infectives [30].

*Rubus ulmifolius* Schott., Rosaceae (Elmleaf blackberry) is a wild shrub native to the Mediterranean. A limited number of published studies that have examined the antibacterial properties of *R. ulmifolius*. For example, Flamini *et al.* [31] identified an anthrone (Rubanthrone A) in the leaves, branches and flowering tops of *R. ulmifolius* that had an MIC of 4,500 µg/mL against *S. aureus* (ATCC 25923). Panizzi *et al.* [32] examined a crude extract of *R. ulmifolius* leaves, flowering tops and branches as well as subsequent extracts of increasing polarity for antibacterial activity using the same *S. aureus* strain and found that the greatest activity was present in the crude extract. The importance of *R. ulmifolius* as a traditional medicine was highlighted in an ethnobotanical field study conducted in rural communities of southern Italy [33]. In particular, the fresh leaves are topically applied with pork fat in the treatment of skin and soft tissue infections (SSTI) and a decoction of the roots is used as a wash to prevent hair loss. In a screening study that followed this fieldwork, ethanolic extracts from 104 Italian plants were assessed for their anti-biofilm potential and extracts from *R. ulmifolius* and nine other species were found to show some promise [28]. Here, we expanded on this work to determine whether bioassay-guided fractionation could be used to isolate a more potent extract of *R. ulmifolius* and whether this extract could limit biofilm formation in genotypically and phenotypically-diverse strains of *S. aureus* to a degree that can be correlated with increased antibiotic susceptibility.

## Results

### Bioassay-guided fractionation of the active constituents

**Figure 1:**
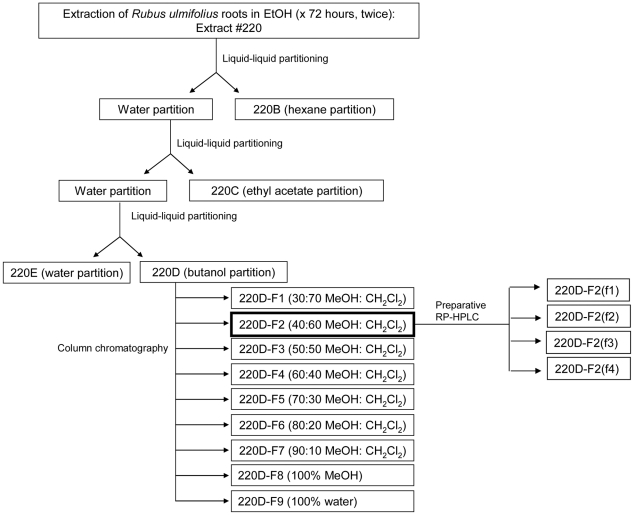
Fractionation scheme for the separation of anti-biofilm constituents found in the roots of *Rubus ulmifolius*.

**Figure 2:**
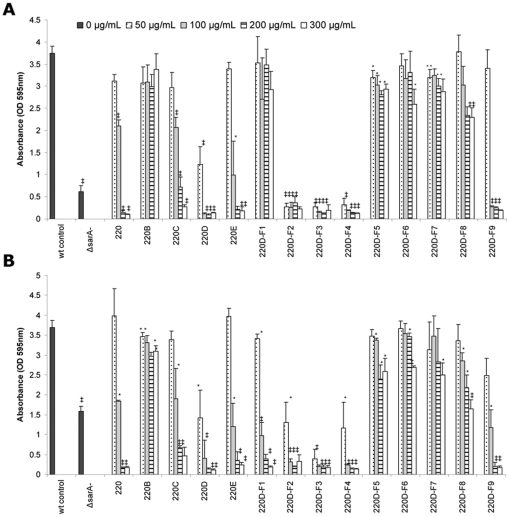
Biofilm inhibition of *R. ulmifolius* root extracts. A static microtiter plate biofilm assay which employed crystal violet as a biofilm matrix staining agent was used to assess the inhibitory activity of individual fractions from the *R. ulmifolius* root using UAMS-1 (**A**) or the USA300 isolate UAMS-1782 (**B**) as the wild-type (wt) test strains. Isogenic *sarA* mutants for each strains were included as controls. Other designations refer to the specific extract as defined by the fractionation scheme illustrated in Figure 1. The MBICs for the crude EtoH extract (220), butanol partition (220D), and 40∶60 (220D-F2), 50∶50 (220D-F3), and 60∶40 (220D-F4) MeOH∶CHCl2 fractions from the butanol partition were 200, 100, 50, 50 and 50 µg/mL, respectively in both UAMS-1 and UAMS-1782. Statistical significance (*, *P*<0.05; ‡, *P*<0.001) refers to differences observed in each parent strain with and without the indicated extract at the indicated concentration.

Fractionation of the crude ethanolic root extract was done as illustrated in Fig. 1. The activity of each fraction was assessed based on the ability to limit biofilm formation in a microtiter plate assay using the methicillin-sensitive (MSSA) strain UAMS-1 and the methicillin-resistant (MRSA) USA300 isolate FPR3757 (UAMS-1782). The greatest degree of inhibition was observed in the butanol partition of the aqueous phase obtained after successive extraction with hexane and ethyl acetate (Fig. 2). Further partitioning of the butanol extract with MeOH∶CH_2_Cl_2_ demonstrated that the greatest activity was observed with fractions ranging from 40∶60 to 60∶40 MeOH∶CH_2_Cl_2_ (220D-F2, 220D-F3 and 220D-F4). In fact, all three of these fractions limited biofilm formation in this assay in a dose dependent manner and to a degree that exceeded that observed with mutation of *sarA* (Fig. 2). Moreover, biofilm assays confirmed that the activity in these extracts was increased by comparison to the crude extract while activity in other MeOH∶CH_2_Cl_2_ extracts was decreased. This was true with both UAMS-1 and UAMS-1782 although at the lowest concentration tested (50 µg/mL) inhibition was slightly greater with the former by comparison to the latter (Fig. 2). While comparable inhibition was observed with the 220D-F2, 220D-F3 and 220D-F4 partitions, overall yield was greatest with 220D-F2 with a percent yield of 0.329% of dry root weight (3.29 g per kg of dry root weight). For this reason, 220D-F2 was selected for subsequent experiments.

**Figure 3:**
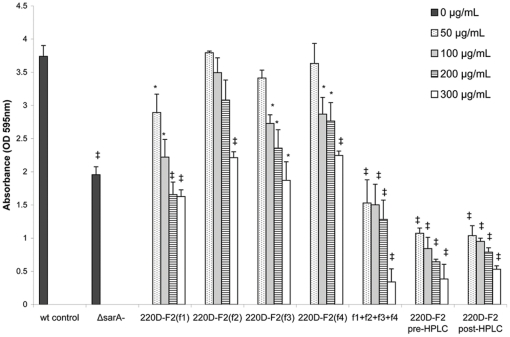
Biofilm inhibition by fractions of 220D-F2. A static crystal violet microtiter plate biofilm assay was used to assess the inhibitory activity of fractions of 220D-F2 both alone and in combination using UAMS-1. No single fraction of 220D-F2 exhibited improved biofilm-inhibiting activity over 220D-F2 as a whole. Multiple combinations of the fractions were made and the only combination which resulted in restoration of activity on the same level as 220D-F2 was when all 4 fractions (f1+f2+f3+f4) were recombined. Likewise, a single collection, in which 220D-F2 was run through the HPLC system and collected as a whole (instead of splitting into fractions) also resulted in the same level of activity as the original 220D-F2. These findings suggest that multiple components found in extract 220D-F2 are necessary for the anti-biofilm activity. Statistical significance (*, *P*<0.05; ‡, *P*<0.001) refers to differences observed in comparison to the untreated control.

A preparative reversed-phase high-performance liquid chromatography (RP-HPLC) method was developed to further separate 220D-F2 into four fractions. Bioassays for biofilm inhibition with these fractions revealed that no single fraction was more effective than 220D-F2 itself, suggesting that more than one fraction was necessary for the anti-biofilm activity (Fig. 3). Thus, additional experiments were conducted in which fractions were combined in all possible permutations and tested for activity (data not shown). The only combination in which activity was restored was when all four fractions were recombined (Fig. 3). These results were also compared with 220D-F2 before and after running through the HPLC system to determine if the separation protocol itself had any effect on the activity. No significant difference between samples could be determined. These data suggest that the anti-biofilm effect of 220D-F2 is due to the synergistic activity of multiple compounds, and that the relative proportions of this mixture is important to the activity.

### Identification of Active Constituents

**Figure 4:**
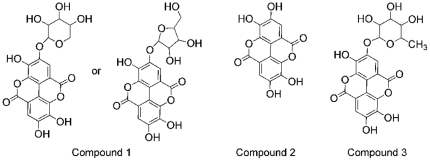
LC-MS/MS analysis revealed a mixture of ellagic acid and glycosylated ellagic acid derivatives in 220D-F2. Corresponding ESI(+)-MS and MS/MS data is reported in Table S1. **Compound 1.** Ellagic Acid xylopyranoside or xylofuanoside **Compound 2.** Ellagic acid. **Compound 3.** Ellagic acid mannopyranoside. The configuration for each of the glycosylated ellagic acids could not be confirmed. Neutral loss of *m/z* 132 was used to confirm the presence of a pentose attached to ellagic acid.

LC-UV/MS/MS (liquid chromatography-ultraviolet absorption-tandem mass spectrometry) studies on 220D-F2 revealed the presence of ellagic acid (EA) and several ellagic acid derivatives (EADs) or sapogenin-related compounds. Molecular formulas of C_14_H_6_O_8_, C_20_H_16_O_12_, C_19_H_14_O_12_, C_30_H_46_O_7_, C_30_H_46_O_8_ (Table 1) were identified and confirmed with accurate mass measurements (<5 ppm). Possible structures for three of these components (C_14_H_6_O_8_, C_20_H_16_O_12_, C_19_H_14_O_12_) are shown in Fig. 4. Neutral loss of a pentose group (*m/z* 132) was observed for peaks 1 (*m/z* 435) and 3 (*m/z* 449). A product ion of *m/z* 303 was observed for both of these peaks suggesting that both components contain an ellagic acid core. A similar product ion was not observed for peaks corresponding to molecular formulae C_30_H_46_O_7_, and C_30_H_46_O_8_ suggesting that these compounds do not contain an ellagic acid core. Likely structures of minor constituents in 220D-F2 could not be determined from these studies.

**Table 1:** Compounds detected in extract 220D-F2 by accurate mass LC/UV/MS/MS.

#	Proposed Compound†	Molecular Formula	Retention Time (minutes)	[M+H]^+^ m/z	MS-MS Fragmentation (*m/z*)
1	Ellagic acid xylopyranoside or Ellagic acid xylofuranoside	C_19_H_14_O_12_	10.9	435.05594	303.01346
2	Ellagic acid	C_14_H_6_O_8_	11.1	303.01354	285.00281, 275.01868, 259.02338, 241.01314
3	Ellagic acid mannopyranoside	C_20_H_16_O_12_	11.1	449.07158	352.33952, 303.01366, 249.11220, 182.98514
4	unknown	unknown	11.5	437.97815	409.09189, 303.01351, 219.10153, 182.98507
5	Sapogenin derivative	C_30_H_46_O_8_	13.5	535.32637	517.31604, 499.30499, 481.29480, 469.29486
6	Sapogenin derivative	C_30_H_46_O_7_	13.7	519.33139 and 501.32097	501.32071, 483.31012, 473.32559, 455.31543, 437.30457, 409.30994
7	unknown	unknown	14.7	573.98540 and 396.98622	532.95864, 505.35274, 485.32649, 451.99400, 440.95009, 352.33958, 317.02929, 273.07586, 199.98796, 182.98517
8	unknown	unknown	16.9	1017.62898 and 999.61893	955.62813, 937.61896, 499.30520, 437.30489

† Proposed structures corresponding to this data are reported in Figure 4. Of the major UV components identified, the most abundant was EA (#2, MW 302). The second most abundant UV component (#7) did not yield a clear MS signal suggestive of a single species or reasonable molecular formula, and thus no structure is proposed. The third most abundant UV component (#1, MW 434) appears to be EA plus a C_5_H_8_O_4_ moiety. A fourth UV component (#3, MW 448) was found to be consistent with a glyosylated derivative of EA. Investigation of the possible formulae consistent with the mass measurement of the fifth UV component (#4) did not yield sufficient data for proposal of a structure. Of the major MS components, the most abundant (#8) did not yield enough information to support the proposal of a structure, however the molecular weights and mass defects suggest that they may be dimers of MW∼500 species (similar to #6). The second most abundant MS component (#6) is consistent with a sapogenin. Successive loss of water (m/z 18) is consistent with a poly-hydroxylated compound. The third most abundant MS component (#5, MW 534) appears to be similar to #6 and has MS/MS losses consistent with a multiply hydroxylated compound like a sapogenin. Losses consisted with neutral loss of a sugar were not observed.

### Prophylactic efficacy of 220D-F2 in limiting *S. aureus* biofilm formation

**Figure 5:**
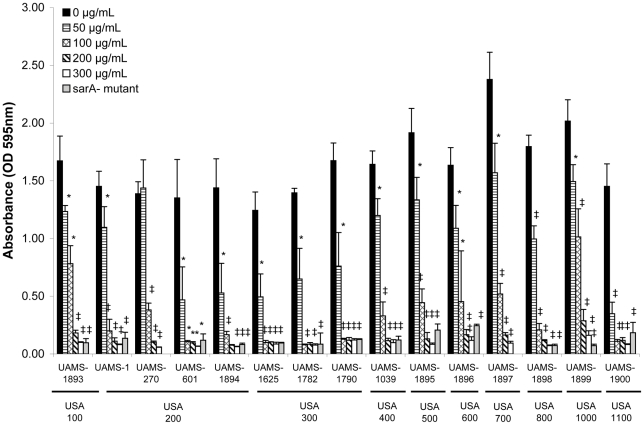
Anti-biofilm activity of 220D-F2 against genotypically- and phenotypically-diverse strains of *S. aureus*. A crystal violet microtiter plate biofilm assay was used to assess the impact of 220D-F2 on biofilm formation. Strain designations are given based on both the corresponding author's culture collection (UAMS) and the clonal lineage of each isolate (USA). Statistical significance (*, *P*<0.05; ‡, *P*<0.001) refers to differences between the untreated cultures and cultures exposed to the indicated concentrations. When available, the isogenic *sarA* mutant for each isolate in the absence of 220D-F2 was included as a control; results obtained with all 15 *sarA* mutants were significantly different from those obtained with the isogenic parent strain (*P*<0.001).

**Figure 6:**
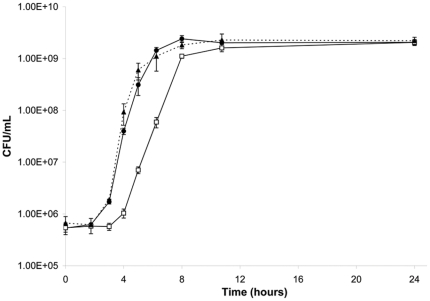
Impact of 220D-F2 on *S. aureus* growth. Results illustrate growth of UAMS-1 in biofilm medium (BM) supplemented with 200 µg/mL 220D-F2 in 0.2% DMSO (□) or 0.2% DMSO (•) as an excipient control. Growth of the isogenic UAMS-1 *sarA* mutant (UAMS-929, containing 0.2% DMSO in comparison with untreated wild type (UAMS-1) and *sar*A mutant (UAMS-929, ▴) is shown for comparison.

The ability of 220D-F2 to limit biofilm formation was assessed against 15 genotypically-diverse clinical isolates of *S. aureus*. The MBIC_90_ for these strains ranged from 50–200 µg/mL (Fig. 5). The MIC and MBC was also determined for all 15 strains, with the MIC_90_ ranging from 530–1040 µg/mL and the MBC_90_ from 530–2040 µg/mL (Table 2). The observation that the MBIC_90_ was at least 2-fold lower than the MIC_90_ and at least 4-fold lower than the MBC_90_ indicates that the inhibition of biofilm formation observed with 220D-F2 was due to something other than growth limitation. This was confirmed by demonstrating that, while the presence of the extract did delay entry into the exponential growth phase by ∼2 hours, cultures with and without 220D-F2 reached the same density within 12 hours and maintained the same cell counts throughout the stationary growth phase (Fig. 6).

**Table 2:** Activity of 220D-F2 (µg/mL) against wild-type strains of *Staphylococcus aureus*.

			Growth		Survival		Biofilm Formation	
USA Type	Strain I.D. of wild type*	Strain I.D. of *sarA* mutant	MIC_50_	MIC_90_	MBC_50_	MBC_90_	MBIC_50_	MBIC_90_
100	UAMS-1893	UAMS-1941	380	530	530	740	100	200
200	UAMS-1	UAMS-929	380	530	740	1040	50	50
	UAMS-270	-	380	530	1460	2040	100	200
	UAMS-601	UAMS-950	530	740	1460	2040	50	100
	UAMS-1894	UAMS-1945	380	530	740	1040	50	100
300	UAMS-1625	UAMS-1653	380	530	530	740	50	100
	UAMS-1782	UAMS-1804	380	530	380	530	50	100
	UAMS-1790	UAMS-1796	380	530	380	530	50	100
400	UAMS-1039	UAMS-1938	530	740	530	740	100	200
500	UAMS-1895	UAMS-1942	740	1040	1040	1460	100	200
600	UAMS-1896	UAMS-1943	530	740	530	1040	100	200
700	UAMS-1897	-	530	740	530	1040	100	200
800	UAMS-1898	UAMS-1944	530	740	1460	2040	100	200
1000	UAMS-1899	UAMS-1930	530	740	1040	1460	100	300
1100	UAMS-1900	UAMS-1931	380	530	740	1460	50	100

*A detailed description of the bacterial strains used in this study has been previously published [52].

**Figure 7:**
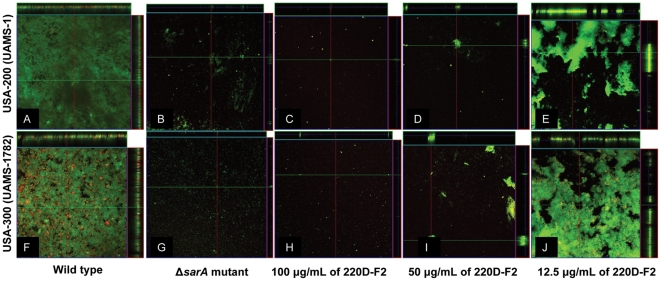
Impact of 220D-F2 as assessed by confocal microscopy. Microtiter plate biofilm assays were undertaken with UAMS-1 (top) or UAMS-1782 (bottom) after the addition of either 220D-F2 at the indicated concentrations or excipient (DMSO) to the growth medium. Confocal images were obtained after 20 hours of incubation. An orthogonal view is included to illustrate overall biofilm architecture at a magnification of 10×. Isogenic *sarA* mutants grown in BM with DMSO were included as negative controls.

The ability of 220D-F2 to inhibit biofilm formation was also confirmed by confocal microscopy (Fig. 7). At concentrations as low as 50 µg/mL, the degree of inhibition observed with 220D-F2 was comparable to that associated with mutation of *sarA* in both UAMS-1 and the USA300 isolate UAMS-1782 at concentrations as low as 50 µg/mL. More specifically, the untreated wild-type strains formed uniform biofilms with a thickness ranging from 88–92 µm while the isogenic *sarA* mutants and parent strains exposed to 220D-F2 formed very patchy biofilms with a few isolated clumps of adherent cells.

### Therapeutic efficacy of 220D-F2 in removal of established *S. aureus* biofilms

The microtiter plate assay used in the experiments discussed above does not lend itself easily to studies assessing relative antibiotic susceptibility. To address this, we employed an *in vitro* model of catheter-associated biofilm formation [34]. In order to assess the impact of 220D-F2 relative to an isogenic *sarA* mutant, we first examined the ability of 220D-F2 to inhibit colonization of the catheters. The total number of viable cells adherent to catheters following exposure to 220D-F2 during colonization was significantly reduced by comparison to the untreated control (*P*<0.05) and comparable to the number observed with the isogenic *sarA* mutant. Although the actual reduction in the number of adherent cells was relatively modest (1.23×10^6^ CFU/catheter in the presence of 220D-F2 versus 1.34×10^7^ CFU/catheter in the untreated control), previous work comparing UAMS-1 with its isogenic *sarA* mutant demonstrated that a similar level of inhibition was sufficient to improve the therapeutic response of a biofilm-associated infection to antimicrobial therapy under both *in vitro* and *in vivo* conditions [35].

**Figure 8:**
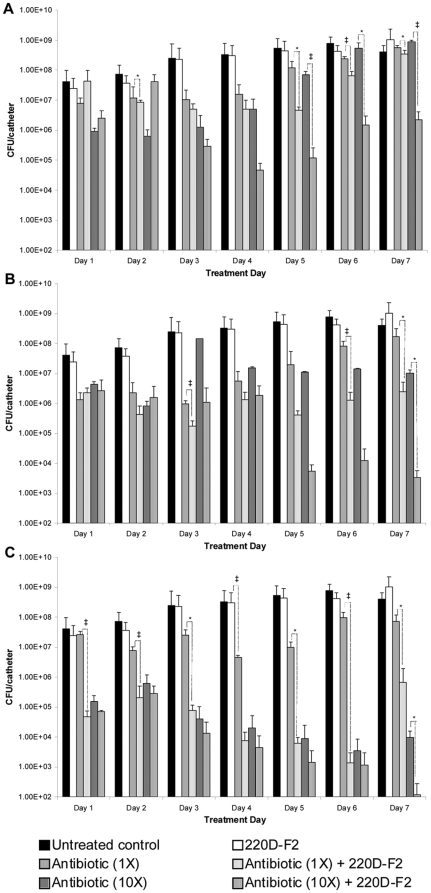
Use of 220D-F2 as adjunct therapy with conventional antibiotics. Biofilms were formed on plasma-coated catheters for 24 hours by growth of the test strain (UAMS-1) in BM. Catheters were then placed in fresh BM containing 200 µg/mL 220D-F2 with or without the indicated amounts of antibiotic. In the case of all three antibiotics, the concentrations examined correspond to 1× or 10× the CLSI-defined breakpoint MIC for a sensitive strain of *S. aureus*. Statistical significance (*, *P*<0.05; ‡, *P*<0.001) refers to differences between the cultures treated with antibiotic alone and cultures exposed to both extract 220D-F2 and antibiotic. **A.** Clindamycin (1×: 0.5 µg/mL; 10×: 5 µg/mL); **B.** Daptomycin (1×: 1 µg/mL; 10×: 10 µg/mL); **C.** Oxacillin (1×: 0.5 µg/mL; 10×: 5 µg/mL).

Based on these results, we used the *in vitro* catheter model to examine the therapeutic efficacy of 220D-F2 with and without concomitant antimicrobial therapy. In the absence of antibiotic, 220D-F2 had no impact on an established biofilm even after 7 consecutive days of exposure (Fig. 8). However, exposure of a UAMS-1 biofilm to 200 µg/mL 220D-F2 concomitantly with different antibiotics (daptomycin, clindamycin, and oxacillin) was shown to offer a significant therapeutic benefit by comparison to antibiotic alone. For example, catheters exposed to 220D-F2 and 10 µg/mL daptomycin (10× breakpoint MIC) exhibited average colony counts of 3.37×10^3^ CFU/catheter while those exposed to daptomycin alone exhibited colony counts of 1.01×10^7^ (Fig. 8). Enhanced efficacy was also observed with clindamycin and oxacillin. For example, exposure to 220D-F2 and clindamycin (10×) for 7 consecutive days resulted in a ∼2.5 log reduction by comparison to antibiotic alone (Fig. 8). Although we did not observe a significant difference between oxacillin and oxacillin+220D-F2 when oxacillin was used at a 10× concentration, when used at a concentration corresponding to the MIC of UAMS-1 (0.5 µg/mL), catheters exposed to 220D-F2 and oxacillin exhibited up to a 2–5 log reduction (depending on the number of days of exposure) by comparison to oxacillin alone (Fig. 8).

**Figure 9:**
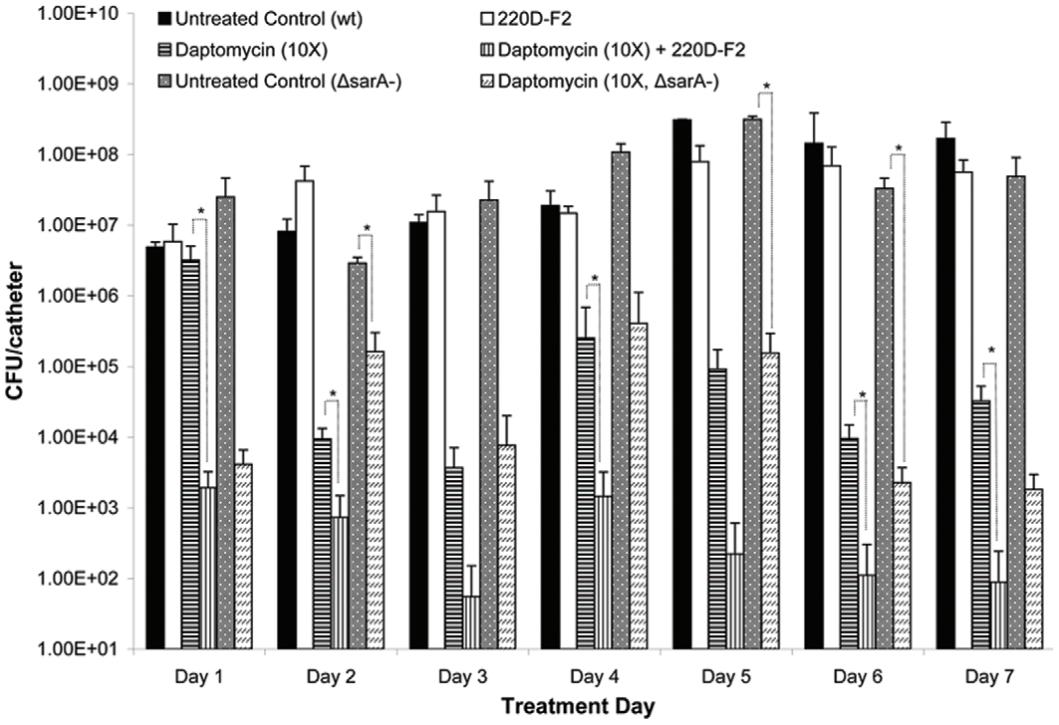
Prophylactic use of 220D-F2 prior to concomitant antibiotic therapy. Biofilms were formed on plasma-coated catheters for 24 hours by growth of the test strain (UAMS-1) in BM containing 200 µg/mL 220D-F2. Catheters were then placed in fresh BM containing 200 µg/mL 220D-F2 with or without 10 µg/mL of daptomycin, which corresponds to 10× the CLSI-defined breakpoint MIC for a sensitive strain of *S. aureus*. Statistical significance (*, *P*<0.05; ‡, *P*<0.001) refers to differences between the untreated cultures and cultures exposed to the compounds. Results observed with the isogenic *sarA* mutant with and without the same concentration of daptomycin but without 220D-F2 are shown for comparison.

An experiment in which 220D-F2 was added to the growth medium during biofilm formation and then included with and without daptomycin (10×) during the treatment phase of the experiment was also undertaken. Colony counts in the group exposed to daptomcyin alone were reduced to 3.37×10^6^ CFU/catheter on day 1 while those in the group exposed to both 220D-F2 and daptomycin were reduced to 1.93×10^3^ CFU/catheter (Fig. 9). This effect was even more apparent after 7 days of exposure, with colony counts on catheters exposed to daptomycin alone falling to 3.27×10^4^ CFU/catheter and those exposed to daptomycin and 220D-F2 falling to 8.87×10^1^. Moreover, ≥33% of all catheters exposed to both 220D-F2 and daptomycin were cleared of all adherent cells (lower detection limit of 10 cells/catheter) beginning on day 2 and continuing on all treatment days thereafter.

### Anti-biofilm activity of select *R. ulmifolius* constituents

Ten commercially available phytochemical constituents reported in the literature [31], [32], [36] to have been isolated from *R. ulmifolius* (Table 3) were purchased and examined for their prophylactic efficacy in the prevention of biofilm formation using a static microtiter plate biofilm assay. The only compound exhibiting meaningful anti-biofilm activity at doses well below any growth inhibitory effects was EA (Table S1). This finding supports the hypothesis that EA and EADs present in 220D-F2 are responsible for the anti-biofilm properties of the extract.

**Table 3:** Phytochemicals previously isolated from *Rubus ulmifolius* [31], [32], [36].

kaempferol-3-O-(6″-*p*-coumaroyl)-β-D-glucopyranoside	*quercetin
kaempferol-3-*O*-α-L-arabinopyranoside	rubanthrone A
kaempferol-3-O-(6″-feruloyl)-β-D-glucopyranoside	rubanthrone B
kaempferol-3-*O*-β-D-galactoside	rubanthrone C
quercetin-3-*O*-β-D-glucuronide	*caffeic acid
quercetin-3-*O*-β-D-glucoside	tormentic acid
quercetin-3-*O*-α-L-rhamnoside	*ursolic acid
*quercetin-3-*O*-glucuronide	euscaphic acid
luteolin-7-*O*-β-D-glucuronide	*oleanolic acid
kaempferol-3-*O*-glucuronide	2α-hydroxyursolic acid
kaempferol-3-*O*-β-D-glucuronide	*ferulic acid
kaempferol-3-*O*-β-D-glucoside	*tiliroside
tormentic acid-28-glucoside	corosine
23-hydroxy tormentic acid	*gallic acid
euscaphic acid-28-glucoside	nigaichigoside
ursolic acid-28-glucoside	3-caffeoylquinic acid
1,4-dicaffeoylquinic acid	5-caffeoylquinic acid
4-caffeoylquinic acid	*kaempferol
*ellagic acid	

*Individual compounds tested for anti-biofilm activity. Results are reported in Table 3.

### Cytotoxicity of 220D-F2 in normal mammalian cell lines

**Figure 10:**
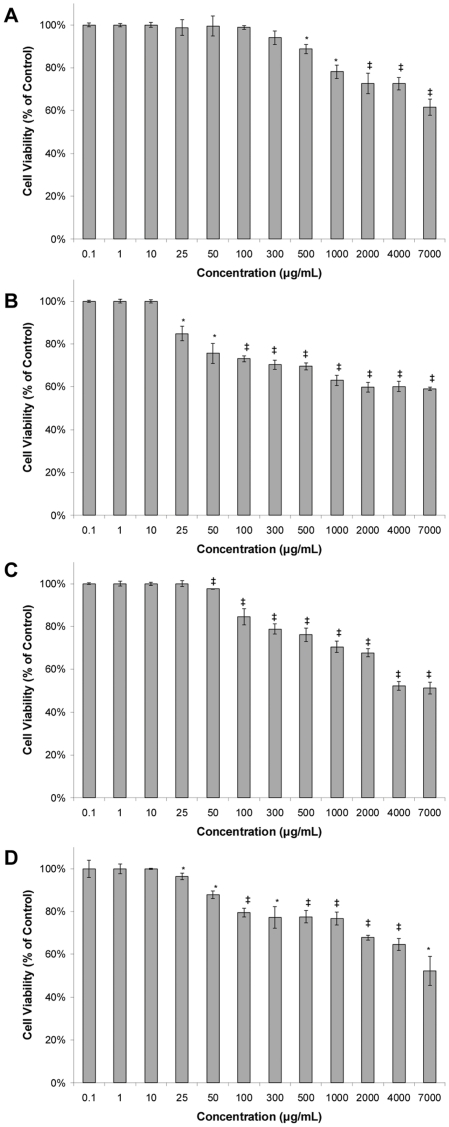
Cytotoxicity of 220D-F2 against normal mammalian cell lines. Cytotoxicity was assessed using a lactate dehydrogenase (LDH) test following 24 hours of exposure to the extract. Results are reported as the percent of cell viability after exposure to the indicated dosage of 220D-F2 in the culture growth medium. Statistical significance (*, *P*<0.05; ‡, *P*<0.001) refers to differences observed in comparison to the untreated (excipient) control. **A.** Normal human kidney proximal tubular (HK-2) cells; **B.** Normal mouse kidney proximal tubular (TKPTS) cells; **C.** Normal rat kidney (NRK-52E) cells; **D.** Normal mouse hepatocytes (AML12).

Normal human (HK-2) and mouse (TKPTS) proximal tubular kidney cells demonstrated very good tolerance for the extract, and no IC_50_ could be identified even at extremely high doses of 7,000 µg/mL (Fig. 10). Rat kidney (NRK-52E) cells and mouse hepatocytes (AML-12) were slightly more sensitive and had IC_50 s_ of 4,000 and 7,000 µg/mL, respectively. Human kidney cells were the least impacted and a significant effect in decreasing cell viability was notable only at concentrations ≥500 µg/mL. These results are relevant as the active doses for biofilm inhibition range from 50–200 µg/mL (depending on the *S. aureus* strain) and no or very limited impact (<20%) on cell viability was notable at these concentrations in the cell lines examined.

## Discussion

In a previous study we demonstrated that an ethanolic extract from the root of *R. ulmifolius* can be used to limit *S. aureus* biofilm formation [28]. However, this work was limited to the crude extract and examined only one strain of *S. aureus*. In this report, we expanded on this work by fractionating the extract and assessing the anti-biofilm properties of the resulting fractions against genotypically and phenotypically-diverse strains of *S. aureus*. The results confirmed that an EA and EAD-rich fraction (220D-F2) obtained from the roots of *R. ulmifolius* was effective at preventing *S. aureus* biofilm formation irrespective of strain identity. While addition of the same fraction had no impact on dispersal of an established biofilm, this limitation was therapeutically relevant in that inclusion of 220D-F2 was associated with enhanced susceptibility to functionally-diverse antibiotics including daptomycin, oxacillin, and clindamycin in the specific context of an established biofilm. Moreover, when the extract was employed both prophylactically during the colonization process and therapeutically in combination with daptomycin, it was possible to completely clear infected catheters of all viable cells.

A few studies [31], [32], [36] have addressed the phytochemical makeup of *R. ulmifolius* and identified some compounds (Table 3), but it is unlikely that this list is comprehensive. Indeed, the EADs isolated in this study have not been previously reported for *R. ulmifolius*. Importantly, we demonstrated that EA alone has potent anti-biofilm properties at concentrations (MBIC_50_<50 µM) well below those that impact bacterial growth (MIC_90_>2000 µM). This is consistent with the hypothesis that the activity of 220D-F2 is likely to be related to the high content of EA and glycosylated EAs. The decline in activity when 220D-F2 is divided into four separate fractions may be related to the EAD content of the various fractions and possibly the unequal distribution of the most active forms. More work involving the isolation of substantive amounts of individual EADs for further testing and structural elucidation by NMR (nuclear magnetic resonance) is necessary to determine which compound(s) are most efficacious in preventing *S. aureus* biofilm formation and if they act in a distinctly synergistic fashion when combined.

**Figure 11:**
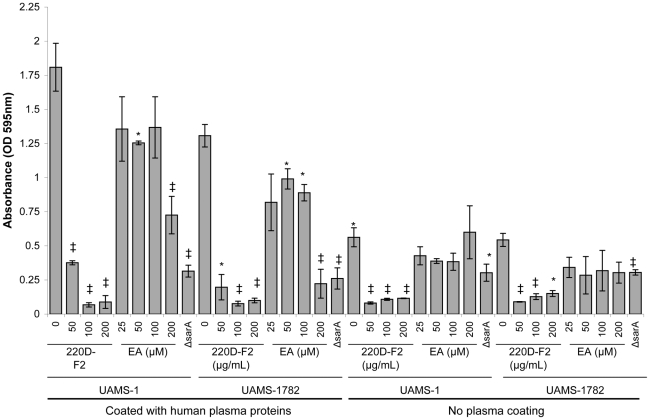
Biofilm inhibition in the presence and absence of human plasma proteins. Previous studies have indicated that ellagic acid is not an effective biofilm inhibitor for *S. aureus* [47]. Our data suggest otherwise. UAMS-1 (USA200) and UAMS-1782 (USA300) were used to assess the efficacy of 220D-F2 and EA in experiments that included or omitted the use of plasma coating for the test wells. Treatment with 220D-F2 elicited a dose-dependent response in limiting biofilm formation under both growth conditions, whereas ellagic acid inhibited biofilm formation only in plasma coated wells, suggesting that the mechanistic basis of EA's biofilm-inhibiting effects involves surface protein recognition or attachment.

EA is a polyphenol found at high concentrations in a number of edible fruits, such as grapes, strawberries, raspberries, blackberries, and black currants. Berries of the Rosaceae family, in particular, contain high levels of EA equivalents [37]. EA is derived from gallic acid, in which two gallic acid molecules are linked by ester bonds. EA has been the focus of many studies in recent years, primarily related to its antioxidant [38], [39], anti-proliferative [40], anti-estrogenic [41] anti-inflammatory [42], anti-bacterial [37], [43] and protein kinase CK-2 inhibiting [44] effects. Reports concerning the biofilm inhibiting properties of EA against *Escherichia coli* [45], [46] *Streptococcus dysgalactiae* [47], *Pseudomonas putida* [46], and *Burkholderia cepacia* [46] have also recently emerged. EADs, on the other hand, have been found to have anti-plasmodial [48], anti-babesial [49], antibacterial [50] and antioxidant [50], [51] effects. However, to our knowledge, this is the first report of the anti-biofilm properties of EA and EADs against *S. aureus*. This is in contrast to Dürig *et al.* [47], who examined the impact of EA on *S. aureus* biofilm formation but did not observe any inhibitory activity. This contrast may be attributable to differences in the methodologies employed in the respective studies. Specifically, we employ in vitro assays in which the substrate, whether microtiter plate or catheter, is first coated with plasma proteins [34], [52]. We do this both because indwelling medical devices are invariably coated with plasma proteins and because the results we have obtained with our *in vitro* assays have been consistent with those obtained using *in vivo* assays [35]. This was not done in the study by Dürig *et al.* [47]. This is potentially important in that we have also demonstrated that the results obtained when examining the contribution of specific *S. aureus* factors to biofilm formation differ depending on whether the assay is done with or without plasma coating [34], [52], [53]. To determine whether this may account for this discrepancy, we tested both 220D-F2 and EA in a biofilm assay with and without plasma coating. Our results confirmed that, when the substrate is coated with plasma proteins, EA elicits a significant decrease in the biofilm phenotype that is not apparent in the absence of coating with plasma proteins (Fig. 11). Thus, it is likely that this difference is related to EA interactions with proteins. At the same time, the inhibitory activity of 220D-F2 was apparent under both conditions, which suggests that 220D-F2 contains components unrelated to EA that also have anti-biofilm properties or that alternative derivatives of EA exist in the extract that are not present in the commercially-available EA preparation.

The focus in this study was on further characterization of the crude *R. ulmifolius* extract and confirmation of its anti-biofilm properties in diverse strains of *S. aureus*. Nevertheless, the results discussed above potentially provide important clues about the mechanistic basis for this activity. Most notably, the fact that the inhibitory effects of 220D-F2 were apparent with and without plasma coating suggests that components within the extract are likely to impact multiple biofilm-associated processes. This also appears to be the case with *sarA* in that the negative impact of mutating *sarA* on biofilm formation is apparent, albeit to greatly varying degrees, irrespective of whether the assay is done with plasma coating [52], [54]. Mutation of *sarA* results in decreased production of the polysaccharide intercellular adhesion (PIA), increased production of extracellular nucleases, and increased production of extracellular proteases [52], [53], [54]. The impact of *sarA* on the production of PIA appears to play a minor role by comparison to these other factors [53], and this suggests that the inhibitory effects we observed with 220D-F2 are not mediated by changes in PIA production. This is consistent with the observation that 220D-F2 did not inhibit biofilm formation in *S. epidermidis* (data not shown). We have demonstrated that the increased production of both nucleases and proteases contribute to the biofilm-deficient phenotype of *sarA* mutants [52], [53], [54]. However, the impact of eliminating nuclease production in a *sarA* mutant is apparent only when the assay is done without plasma coating. To the extent that 220D-F2 inhibited biofilm formation both with and without plasma coating, it is plausible to suggest that the inhibitory effects may impact both protein-dependent and protein-independent mechanisms of biofilm formation in much the same way as *sarA* itself. We included *sarA* mutants as controls based on previous work demonstrating that mutation of *sarA* limits biofilm formation to a therapeutically-relevant degree [34], [35], but based on this we also examined whether the presence of 220D-F2 had any impact on expression of *sarA*, and somewhat surprisingly our results to date suggest that this is not the case (data not shown). Nevertheless, the results reported in this study provide strong support for the hypothesis that 220D-F2 or its associated components may well have therapeutic utility in the specific context of an *S. aureus* biofilm-associated infection.

## Materials and Methods

### Acquisition of botanical materials

Bulk samples of *Rubus ulmifolius* Schott. (Rosaceae) roots were collected from wild populations in August 2009 in the village of Ginestra, Italy. Procedures from the 2003 WHO Guidelines on Good Agricultural and Collection Practices for Medicinal Plants [55] were followed for the collection and identification of bulk and voucher specimens. Voucher specimens (CQ-164) were deposited at the *Herbarium Lucanum* (HLUC) at the *Università della Basilicata* in Potenza, Italy. Additional vouchers are in the possession of the first author. The specimens were identified using the standard Italian Flora [56] and identification was confirmed at HLUC. All soil and other contaminants (i.e. insects, other plant species etc.) were removed from each sample. Roots were cut into small pieces and air dried. Upon drying, materials were packed into plastic bags with silica packets and vacuum sealed, and then exported to the USA under USDA Permit PDEP-09-00228 for phytochemical evaluation and bioassays.

### Extraction and bioassay guided fractionation of plant materials

Air-dried roots (1 kg) were ground into a fine powder and extracted with 95% EtOH (2×10 L) at room temperature for 72 hours with constant agitation. Filtered extracts were combined, concentrated at reduced pressure and a temperature <45°C, and lyophilized before being re-suspended in water and partitioned in succession with hexane, ethyl-acetate and butanol (all solvents acquired from Fisher Chemical, Certified ACS). The partitions were dried over anhydrous sodium sulfate, concentrated at reduced pressure, and lyophilized before testing for activity. The most active partition (butanol) was subjected to column chromatography using Silica gel (0.015–0.040 µm particle size, EMD Chemicals) and fractions were collected after eluting successively with mixtures of MeOH∶CH_2_Cl_2_ (30∶70, 40∶60, 50∶50, 60∶40, 70∶30, 80∶20, 90∶10) followed by 100% MeOH and 100% H_2_O (18Ω). Fractions were dried, weighed and tested for anti-biofilm activity using an established microtiter plate assay (see below).

A preparative HPLC method was developed to split 220D-F2 into 4 additional fractions in an effort to identify the active constituent(s). Briefly, 220D-F2 was dissolved in 2-propanol∶water (2∶8) at a concentration of 50 mg/mL. A C-18 column (μBondapak™, 19 mm×300 mm, 125 ′Å, 10 µm) was used to separate 1 mL injections (total 5 mg) with a Waters 600E system controller and pump and an isocratic mobile phase of H_2_O∶acetonitrile∶2-propanol∶formic acid (74∶17∶8∶1) at a flow rate of 7 mL/min (all solvents HPLC-grade, Fisher Chemical) and detection at a wavelength of 360 nm using a Waters 486 Tunable Absorbance Detector and Shimadzu C-R5A Chromatopac. Fractions were collected at 17.5 (220D-F2-f1), 20.5 (-f2), 27 (-f3) and 45 (-f4) minutes. The largest single peaks were located in fractions 2 and 3, whereas fractions 1 and 4 contained multiple minor peaks. Fractions were collected for chemical analysis and bioassays described below.

### Characterization of major extract components

Further characterization of 220D-F2 (suspended in 5% isopropanol in H_2_O, 1 mg/mL) was performed using accurate mass LC/UV/MS/MS to identify the major components. A Mac-Mod HALO C18 column (3.0×100 mm) was used with a mobile phase (A: 0.1% formic acid in water; B: 0.1% formic acid in acentonitrile) with a gradient (hold 2% B for 2 min., 2–50% B over 18 min, 100% B for 5 min.), flow of 0.4 mL/min and PDA (photodiode array detector) detection range of 200–790 nm. MS detection was with a Thermo LTQ Orbitrap Discovery, +ESI mode, and scan range of 140–2000 Da. Searches of multiple databases (Human Metabolome Database (version 2.5), ChemSpider, SciFinder, and Kyoto Encyclopedia of Genes and Genomes (KEGG) LIGAND Database) were performed using chemical formula queries. All mass measurements were within 0.5 mmu of the proposed formulae, well within the expected measurement tolerances of the mass spectrometer.

### Individual phytochemicals tested

In addition to testing the chemically complex *R. ulmifolius* extracts for anti-biofilm activity, all known constituents of *R. ulmifolius* (Table 2) that were commercially available were purchased for anti-biofilm analysis using the static microtiter plate method described below. Ferulic acid, kaempferol, ursolic acid, quercetin dehydrate, caffeic acid, ellagic acid, and oleanolic acid were purchased from MP BioMedicals (Solon, OH, USA); quercetin-3-O-glucuronide and tiliroside from Chromadex (Irvine, CA, USA); and gallic acid from Acros Organics (NJ, USA). Compounds were tested for growth and biofilm inhibitory activity at doses ranging from 25–2000 µM.

### Quality control

Extracts and drugs were suspended in either DMSO or PBS (depending on solubility), then sterile filtered (0.2 µm), and stored in sterile vials prior to use in all bioassays. Sterility controls were included in all assays (extract+media). Tests for sterility followed standard quality control methods [57] to ensure that no microbial growth was detectable prior to bioassay testing. Prior to performing bioassays, batches of extract 220D-F2 were checked for the relative ratios of constituents by RP-HPLC, and were found to be reproducible. All chemicals (including extract) were stored at −20°C.

### Bacterial strains and growth conditions

The *Staphylococcus aureus* strains used in these experiments are listed in Table 1. When available, isogenic strains carrying a mutation in the staphylococcal accessory regulator (*sarA*) were included as negative controls based on the observation that mutation of *sarA* results in a reduced capacity to form a biofilm that can be correlated with increased antibiotic susceptibility [35], [53]. For biofilm assays, strains were grown in tryptic soy broth (TSB) supplemented with 3.0% NaCl (wt/vol) and 0.5% dextrose (biofilm medium, BM). In experiments that included daptomycin, BM was supplemented (wt/vol) with 2.5 mM CaCl_2_. For all assays, overnight cultures of the test strains were used to inoculate fresh medium at an initial cell density of 5×10^5^ colony-forming units (CFU) per ml (confirmed with plate counts). This cell density was achieved by taking the optical density of overnight cultures and diluting to an OD_560 nm_ of 0.05. Studies examining growth rate were done at 37°C in BM with constant shaking (200 rpm) and a volume-to-flask ratio of 0.4.

### Determination of MIC and MBC

To determine the minimum inhibitory concentration (MIC) and minimum bactericidal concentration (MBC) for each fraction, strains were grown at 37°C in cation-adjusted Mueller-Hinton broth (CAMHB). MIC and MBC were determined following Clinical and Laboratory Standards Institute (CSLI) broth microdilution guidelines [58], [59]. Briefly, test strains were inoculated into 0.1 ml CAMHB containing varying concentrations of extract. For MIC, optical density (OD_600_) was assessed immediately after inoculation and again after 18 hours using a Biotek Synergy II microplate reader. Corrections for extract color were done as previously described [28]. The MIC was defined as the lowest concentration that inhibited growth to a level ≥90% (for MIC_90_) or ≥50% (for MIC_50_) by comparison to untreated control cultures. MBC was assessed by determining the number of colony-forming units (CFU) after 24 hours of exposure and was defined as the lowest concentration at which the initial density of viable cells was reduced to a level by ≥90% (for MBC_90_) or ≥50% (for MBC_50_) by comparison to the untreated control cultures.

### Assessment of biofilm formation

The primary biofilm assays, used as a guide during fractionation, were undertaken using a human plasma protein-coated microtiter plate assay as previously described [53]. Wells were pre-coated for 24 h at 4°C with 20% human plasma diluted in carbonate buffer (pH 9.6). After inoculation and addition of the appropriate media (containing drug or excipient alone), the plates were incubated without shaking at 37°C for 24 h, the wells were gently washed twice with 200 µl of phosphate-buffered saline to remove nonadherent cells. Adherent biofilms were fixed with 200 µl of 100% ethanol prior to staining for 2 min with 200 µl of 0.41% (wt/vol) crystal violet in 12% ethanol (Protocol Crystal Violet; Biochemical Sciences, Swedesboro, N.J.). The stain was then aspirated, and the wells were washed several times with phosphate-buffered saline. A quantitative assessment of biofilm formation was obtained by adding 100 µl of 100% ethanol and incubating at room temperature for 10 min. A total of 50 µl of the eluate was then transferred to a sterile polystyrene microtiter plate and the absorbance (OD_595 nm_) was determined using a plate reader. The minimum biofilm-inhibiting concentration (MBIC) was defined as the lowest concentration of extract in which biofilm formation was limited to a level ≥90% (for MBIC_90_) or ≥50% (for MBIC_50_) by comparison to the untreated parent control strain. Additional tests with 220D-F2 and EA were conducted to examine the influence of plasma proteins on the relative activity of the drugs. In this case, both coated and uncoated wells were employed in the static microtiter plate assay.

Assays examining the impact of different extracts on antibiotic susceptibility were done using an *in vitro* model of catheter-associated biofilm formation [34]. Briefly, 1-cm segments of fluorinated ethylene propylene catheters (14-gauge Introcan Safety catheter; B. Braun, Bethlehem, PA) were coated with human plasma proteins (Sigma-Aldrich, St. Louis, MO, USA), placed in the wells of a 12-well microtiter plate containing BM, and inoculated with the test strain at an initial optical density (OD_560 nm_) of 0.05. After 24 hours, the BM was replaced with fresh BM containing 220D-F2 (or excipient alone) with or without antibiotic. Antibiotics tested were daptomycin, clindamycin, and oxacillin, all of which were examined at concentrations corresponding to 1 and 10 times (1× and 10×) the CLSI-defined breakpoint MIC defined for an antibiotic-resistant strain of *S. aureus*. The medium was replaced in its entirety at 24 hour intervals for 7 days. Following each 24 hour interval, catheters were removed and processed to assess viability as previously described [34], [35]. More specifically, catheters were dunked into sterile PBS several times using a sterile forceps in order to remove all of the loosely adherent cells from both the interior lumen of the catheter and external surface. In a second set of experiments, extracts were added to BM during colonization of plasma-coated catheters. After 24 hours, catheters were either processed for plate counts as described above or transferred to fresh BM with and without antibiotics.

### Confocal laser scanning microscopy (CLSM) of static biofilms

Two *S. aureus* strains (UAMS-1 and the USA300 isolate UAMS-1782) and their isogenic *sarA* mutants (UAMS-929 and UAMS-1804) were grown in 96 well microtiter plates (Costar 3603, Corning Life Sciences) as described above. After 20 hours, the well contents were aspirated and the wells gently washed three times with 0.85% (wt/vol) NaCl. The adherent biofilm was then stained with LIVE/DEAD stain (Invitrogen) at room temperature in the dark for 18 minutes. After removal of the stain, the wells were gently washed with 0.85% NaCl before collecting CLSM images using a Zeiss LSM 510 Meta confocal scanning system and inverted microscope. SYTO 9 fluorescence was detected by excitation at 488 nm and emission collected with a 500–530 bandpass filter. All z-sections were collected at 4-µm intervals using a 10× objective lens. A 0.9×0.9 mm section of biofilm was selected from the center of the well for each image. Image acquisition and processing was performed using LSM Image Browser (Carl Zeiss). Identical acquisition settings were employed for all samples.

### Cell culture and cytotoxicity assays

Normal human kidney proximal tubular (HK-2) cells, normal rat kidney (NRK-52E) cells, and normal mouse hepatocytes (AML12) were purchased from the American Type Culture Collection (ATCC). Normal mouse kidney proximal tubular cells (TKPTS) were developed and gifted by Dr. Elsa Bello-Reuss [60]. The four cell lines were cultured with different media, keratinocyte serum free media (K-SFM) supplemented with bovine pituitary extract (BPE) and human recombinant epidermal growth factor (EGF) for HK-2 cells, Dubelcco's Modified Eagle's Medium (DMEM) for NRK-52E cells, and ATCC complete growth medium (1∶1 mix of DMEM and Ham's F12 medium supplemented with insulin, transferring, dexamethasone, and fetal bovine serum) for AML12 and TKPTS cells. Cells were maintained in humidified air with 5% CO_2_ at 37°C. Cells were transferred to 96-well cell culture plates (10,000 cells seeded per well) and incubated for 24 hours prior to aspirating the media, adding extract 220D-F2 in serum-free media and undertaking cytotoxicity tests. To examine the cytotoxic effects of 220D-F2 on normal mammalian liver and kidney cells, a lactate dehydrogenase (LDH) assay was employed. LDH is a stable cytosolic enzyme that is released upon membrane damage in necrotic cells. LDH activity can serve as a useful measure for determining drug toxicity to cell lines.

LDH was measured using a commercial cytotoxicity assay kit (Promega CytoTox 96® Non-Radioactive Cytotoxicity Assay, WI, USA), in which LDH released in culture supernatants is measured with a coupled enzymatic assay, resulting in conversion of a tetrazolium salt into a red formazan product. The cells were treated with concentrations of extract 220D-F2 ranging from 0.1–7,000 µg/mL and incubated in humidified air with 5% CO_2_ at 37°C for 24 hours. Controls for the extract excipient (20% DMSO in phosphate buffered saline, PBS), positive LDH control, and positive LDH control with media and extract were also included. The sample solution (supernatant) was removed, and the LDH released from the cells into culture medium treated according to kit instructions, then measured at an OD_490 nm_. The maximal release was obtained after treating cells with a lysis solution for 45 minutes, then treating the supernatant according to kit instructions. All tests were performed in quadruplicate. The necrotic percentage (% cytotoxicity) was expressed using the formula: (sample value/maximal release)×100%.

### Statistical analysis

Pair-wise testing was performed based on *t* tests as formatted in Sigma Stat® Statistical Software Version 2 (SPSS, Inc) with *P* values<0.05 considered significant.

## Supporting Information

Table S1 **Inhibitory effects of individual phytochemicals reported in the literature for** ***R. ulmifolius*** **against biofilm formation and growth of UAMS-1.**
(DOCX)

## References

[1] Yamamoto T, Nishiyama A, Takano T, Yabe S, Higuchi W (2010). Community-acquired methicillin-resistant *Staphylococcus aureus*: community transmission, pathogenesis, and drug resistance.. Journal of Infection and Chemotherapy.

[2] Hildron AI, Low CE, Honig EG, Blumberg HM (2009). Emergence of community-acquired methicillin-resistant *Staphylococcus aureus* strain USA300 as a cause of necrotising community-onset pneumonia.. The Lancet Infectious Diseases.

[3] Klevens RM, Morrison M, Nadle J, Petit S, Gershman K (2007). Invasive Methicillin-Resistant *Staphylococcus aureus* infections in the United States.. JAMA.

[4] Harro JM, Peters BM, O'May GA, Archer N, Kerns P (2010). Vaccine development in *Staphylococcus aureus*: taking the biofilm phenotype into consideration.. FEMS Immunology and Medical Microbiology.

[5] Thomas JG, Litton I, Rinde H, Pace JL, Rupp ME, Finch RG (2006). Economic impact of biofilms on treatment costs.. Biofilms, Infection and Antimicrobial Therapy.

[6] Zimmerli W, Waldvogel FA, Vaudaux P, Nydegger UE (1982). Pathogenesis of foreign body infection: description and characteristics of an animal model.. Journal of Infectious Diseases.

[7] Toms AD, Davidson D, Masri BA, Duncan CP (2006). The management of peri-prosthetic infection in total joint arthroplasty.. The Journal of Bone and Joint Surgery.

[8] Brause BD, Mandell GL, Bennett JE, Dolin R (2005). Infections with prostheses in bones and joints.. Principles and Practices of Infectious Diseases.

[9] Wolcott RD, Rhoads DD, Bennett ME, Gogokhia L, Costerton JW (2010). Chronic wounds and the medical biofilm paradigm.. Journal of Wound Care.

[10] Wolcott RD, Dowd SE (2010). The role of biofilms: are we hitting the right target?. Plastic and Reconstructive Surgery.

[11] Kalan L, Wright GD (2011). Antibiotic adjuvants: multicomponent anti-infective strategies.. Expert Reviews in Molecular Medicine.

[12] Cox PA, Balick MJ (1994). The ethnobotanical approach to drug discovery.. Scientific American.

[13] Howell A, Botto H, Combescure C, Blanc-Potard A-B, Gausa L (2010). Dosage effect on uropathogenic Escherichia coli anti-adhesion activity in urine following consumption of cranberry powder standardized for proanthocyanidin content: a multicentric randomized double blind study.. BMC Infectious Diseases.

[14] Howell AB (2007). Bioactive compounds in cranberries and their role in prevention of urinary tract infections.. Molecular Nutrition & Food Research.

[15] Pérez-López FR, Haya J, Chedraui P (2009). Vaccinium macrocarpon: An interesting option for women with recurrent urinary tract infections and other health benefits.. Journal of Obstetrics and Gynaecology Research.

[16] Low CF, Chong PP, Yong PVC, Lim CSY, Ahmad Z (2008). Inhibition of hyphae formation and SIR2 expression in *Candida albicans* treated with fresh *Allium sativum* (garlic) extract.. Journal of Applied Microbiology.

[17] Bodini SF, Manfredini S, Epp M, Valentini S, Santori F (2009). Quorum sensing inhibition activity of garlic extract and p-coumaric acid.. Letters in Applied Microbiology.

[18] Harjai K, Kumar R, Singh S (2010). Garlic blocks quorum sensing and attenuates the virulence of *Pseudomonas aeruginosa*.. FEMS Immunology & Medical Microbiology.

[19] Fulghesu L, Giallorenzo C, Savoia D (2007). Evaluation of Different Compounds as Quorum Sensing Inhibitors in *Pseudomonas aeruginosa*.. Journal of Chemotherapy.

[20] Smith ECJ, Kaatz GW, Seo SM, Wareham N, Williamson EM (2007). The phenolic diterpene Totarol Inhibits multidrug efflux pump activity in *Staphylococcus aureus*.. Antimicrob Agents Chemother.

[21] Chérigo L, Pereda-Miranda R, Fragoso-Serrano M, Jacobo-Herrera N, Kaatz GW (2008). Inhibitors of bacterial multidrug efflux pumps from the resin glycosides of *Ipomoea murucoides*.. Journal of Natural Products.

[22] Wang W, Zeng YH, Osman K, Shinde K, Rahman M (2010). Norlignans, acylphloroglucinols, and a dimeric xanthone from *Hypericum chinense*.. Journal of Natural Products.

[23] Gibbons S (2004). Anti-staphylococcal plant natural products.. Natural Product Reports.

[24] Gibbons S (2008). Phytochemicals for bacterial resistance–strengths, weaknesses and opportunities.. Planta Medica.

[25] Gibbons S, Ohlendorf B, Johnsen I (2002). The genus *Hypericum*–a valuable resource of anti-Staphylococcal leads.. Fitoterapia.

[26] Giusti EM, Pieroni A, Quave CL, Paládi-Kovács A, Csukás G, Kiss R, Kristóf I, Nagy I (2004). Medical anthropology at the borders: ritual healing in Arbëreshë Albanian ethnic communities in Lucania (southern Italy).. Times, Places, Passages Ethnological Approaches in the New Millennium 7th SIEF Selected Papers.

[27] Quave CL, Plano LWR, Bennett BC (2010). Quorum sensing inhibitors for MRSA from Italian medicinal plants.. Planta Medica.

[28] Quave CL, Plano LWR, Pantuso T, Bennett BC (2008). Effects of extracts from Italian medicinal plants on planktonic growth, biofilm formation and adherence of methicillin-resistant *Staphylococcus aureus*.. Journal of Ethnopharmacology.

[29] Lin M-H, Chang F-R, Hua M-Y, Wu Y-C, Liu S-T (2011). Inhibitory effects of 1,2,3,4,6-Penta-O-Galloyl-β-D-Glucopyranose on biofilm formation by *Staphylococcus aureus*.. Antimicrobial Agents and Chemotherapy.

[30] Alviano DS, Alviano CS (2009). Plant Extracts: Search for New Alternatives to Treat Microbial Diseases Current Pharmaceutical Biotechnology.

[31] Flamini G, Catalano S, Caponi C, Panizzi L, Morelli I (2002). Three anthrones from *Rubus ulmifolius*.. Phytochemistry.

[32] Panizzi L, Caponi C, Catalano S, Cioni PL, Morelli I (2002). In vitro antimicrobial activity of extracts and isolated constituents of *Rubus ulmifolius*.. Journal of Ethnopharmacology.

[33] Quave CL, Pieroni A, Bennett BC (2008). Dermatological remedies in the traditional pharmacopoeia of Vulture-Alto Bradano, inland southern Italy.. Journal of Ethnobiology and Ethnomedicine.

[34] Weiss EC, Spencer HJ, Daily SJ, Weiss BD, Smeltzer MS (2009). Impact of sarA on antibiotic susceptibility of *Staphylococcus aureus* in a catheter-associated *in vitro* model of biofilm formation.. Antimicrobial Agents and Chemotherapy.

[35] Weiss EC, Zielinska A, Beenken KE, Spencer HJ, Daily SJ (2009). Impact of sarA on daptomycin susceptibility of *Staphylococcus aureus* biofilms *in vivo*.. Antimicrobial Agents and Chemotherapy.

[36] Dall'Acqua S, Cervellati R, Loi MC, Innocenti G (2008). Evaluation of in vitro antioxidant properties of some traditional Sardinian medicinal plants: Investigation of the high antioxidant capacity of *Rubus ulmifolius*.. Food Chemistry.

[37] Landete JM (2011). Ellagitannins, ellagic acid and their derived metabolites: A review about source, metabolism, functions and health.. Food Research International.

[38] Zafrilla P, Ferreres F, Tomas-Barberan FA (2001). Effect of processing and storage on the antioxidant ellagic acid derivatives and flavonoids of red raspberry (*Rubus idaeus*) jams.. Journal of Agricultural and Food Chemistry.

[39] Lee J-H, Talcott ST (2003). Fruit maturity and juice extraction influences ellagic acid derivatives and other antioxidant polyphenolics in muscadine grapes.. Journal of Agricultural and Food Chemistry.

[40] Losso JN, Bansode RR, Trappey A, Bawadi HA, Truax R (2004). In vitro anti-proliferative activities of ellagic acid.. The Journal of Nutritional Biochemistry.

[41] Papoutsi Z, Kassi E, Tsiapara A, Fokialakis N, Chrousos GP (2005). Evaluation of estrogenic/antiestrogenic activity of ellagic acid via the estrogen receptor subtypes ERα and ERβ.. Journal of Agricultural and Food Chemistry.

[42] Umesalma S, Sudhandiran G (2010). Differential inhibitory effects of the polyphenol ellagic acid on inflammatory mediators NF-κB, iNOS, COX-2, TNF-α, and IL-6 in 1,2-dimethylhydrazine-induced rat colon carcinogenesis.. Basic & Clinical Pharmacology & Toxicology.

[43] Weidner-Wells MA, Altom J, Fernandez J, Fraga-Spano SA, Hilliard J (1998). DNA gyrase inhibitory activity of ellagic acid derivatives.. Bioorganic & Medicinal Chemistry Letters.

[44] Cozza G, Bonvini P, Zorzi E, Poletto G, Pagano MA (2006). Identification of ellagic acid as potent inhibitor of protein kinase CK2: a successful example of a virtual screening application.. Journal of Medicinal Chemistry.

[45] Hancock V, Dahl M, Vejborg RM, Klemm P (2010). Dietary plant components ellagic acid and tannic acid inhibit *Escherichia coli* biofilm formation.. Journal of Medical Microbiology.

[46] Huber B, Eberl L, Feucht W, Polster J (2003). Influence of polyphenols on bacterial biofilm formation and quorum-sensing.. Zeitschrift für Naturforschung.

[47] Dürig A, Kouskoumvekaki I, Vejborg R, Klemm P (2010). Chemoinformatics-assisted development of new anti-biofilm compounds.. Applied Microbiology and Biotechnology.

[48] Simões-Pires CA, Vargas S, Marston A, Ioset JR, Paulo MQ (2009). Ellagic acid derivatives from *Syzygium cumini* stem bark: investigation of their antiplasmodial activity.. Natural Product Communications.

[49] Elkhateeb A, Subeki TK, Matsuura H, Yamasaki M, Yamato O (2005). Anti-babesial ellagic acid rhamnosides from the bark of *Elaeocarpus parvifolius*.. Phytochemistry.

[50] Atta-Ur-Rahman NFN, Choudhary MI, Malik S, Makhmoor T, Nur-E-Alam M (2001). New antioxidant and antimicrobial ellagic acid derivatives from *Pteleopsis hylodendron*.. Planta Medica.

[51] Matthew S, Kao KC, Chang YS, Abreu P (2007). Ellagic acid glycosides from *Turpinia ternata*.. Natural Product Research.

[52] Beenken KE, Mrak LN, Griffin LM, Zielinska A, Shaw LN (2010). Epistatic relationships between sarA and agr in Staphylococcus aureus biofilm formation.. PLoS ONE.

[53] Beenken KE, Blevins JS, Smeltzer MS (2003). Mutation of *sarA* in *Staphylococcus aureus* limits biofilm formation.. Infection and Immunity.

[54] Tsang LH, Cassat JE, Shaw LN, Beenken KE, Smeltzer MS (2008). Factors contributing to the biofilm-deficient phenotype of *Staphylococcus aureus* sarA mutants.. PLoS One.

[55] WHO (2003). WHO guidelines on good agricultural and collection practices (GACP) for medicinal plants.. Geneva.

[56] Pignatti S (2002). Flora d'Italia.

[57] Isenberg HD, Isenberg HD (2004). Clinical Microbiology Procedures Handbook;.

[58] NCCLSNCCLS, editor (1999). Methods for Determining Bactericidal Activity of Antimicrobial Agents; Approved Guideline;.

[59] NCCLS (2001). Methods for Dilution Antimicrobial Susceptibility Tests for Bacteria that Grow Aerobically; Approved Standard.

[60] Ernest S, Bello-Reuss E (1995). Expression and function of P-glycoprotein in a mouse kidney cell line.. American Journal of Physiology - Cell Physiology.

