# Supplementary material for: Ellagic Acid Derivatives from Rubus ulmifolius Inhibit Staphylococcus aureus Biofilm Formation and Improve Response to Antibiotics
Source: PLoS One. 2012 Jan 5;7(1):e28737. doi: 10.1371/journal.pone.0028737 (PMC3252291; doi:10.1371/journal.pone.0028737)
Supplement: Table S1 — Inhibitory effects of individual phytochemicals reported in the literature for R. ulmifolius against biofilm formation and growth of UAMS-1. (DOCX) [file pone.0028737.s001.docx]

**Table S1. Inhibitory effects of individual phytochemicals reported in the literature for *R. ulmifolius* against biofilm formation and growth of UAMS-1.**

|  |  | **Biofilm Formation** | | **Growth** | |
| --- | --- | --- | --- | --- | --- |
| **Chemical Name [CAS #]** | **Structure** | **IC_50_ (μM)** | **IC_90_ (μM)** | **MIC_50_ (μM)** | **MIC_90_ (μM)** |
| Caffeic Acid [331-39-5] | 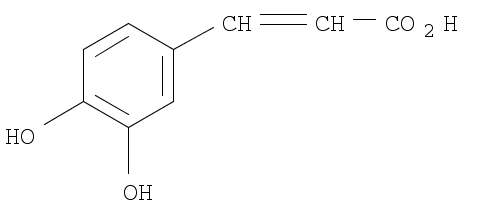 | >2000 | >2000 | >2000 | >2000 |
| Ellagic Acid [476-66-4] | 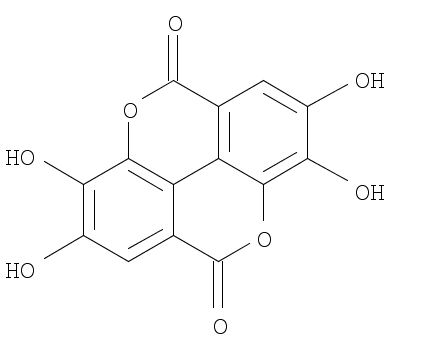 | 50 | 800 | >2000 | >2000 |
| Ferulic Acid [1135-24-6] | 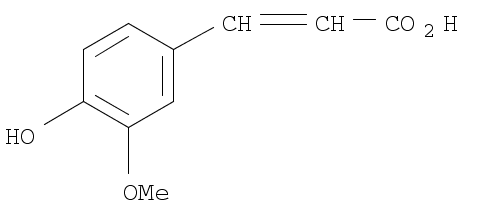 | >2000 | >2000 | >2000 | >2000 |
| Gallic Acid [149-91-7] | 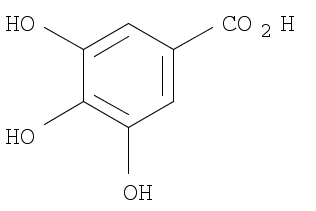 | 800 | >2000 | >2000 | >2000 |
| Kaempferol [520-18-3] | 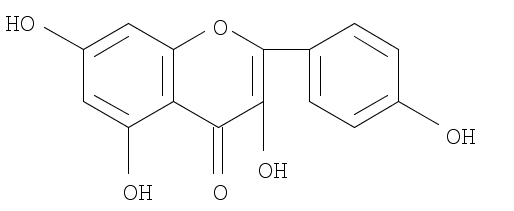 | 600 | 800 | 1700 | 1800 |
| Oleanolic Acid [508-02-1] | 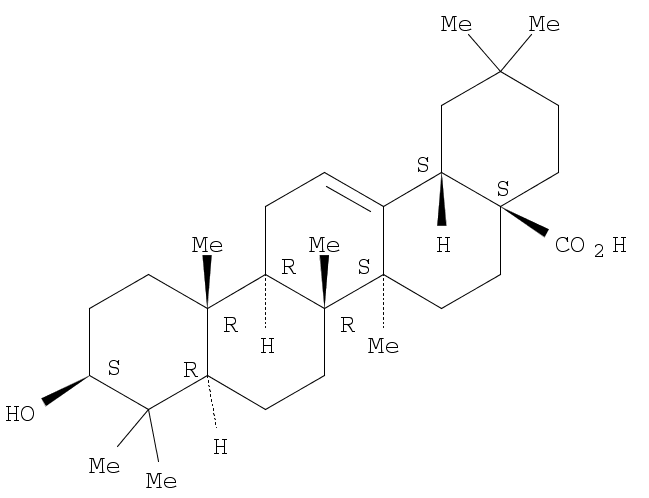 | 200 | 1000 | 1100 | 1600 |
| Quercetin Dihydrate [6151-25-3] | 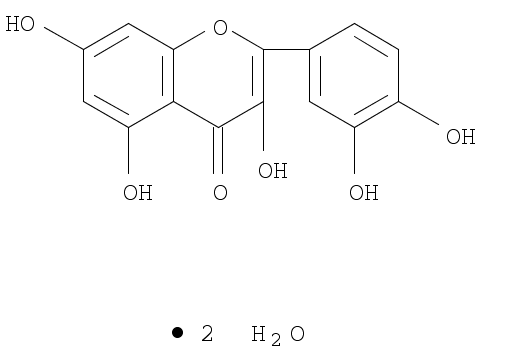 | 600 | 2000 | 1700 | 1900 |
| Quercetin-3-O-glucuronide [22688-79-5] | 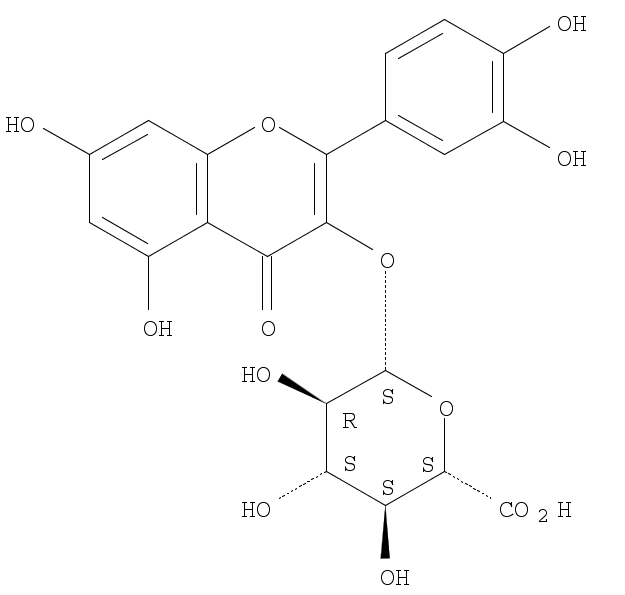 | >2000 | >2000 | >2000 | >2000 |
| Tiliroside [20316-62-5] | 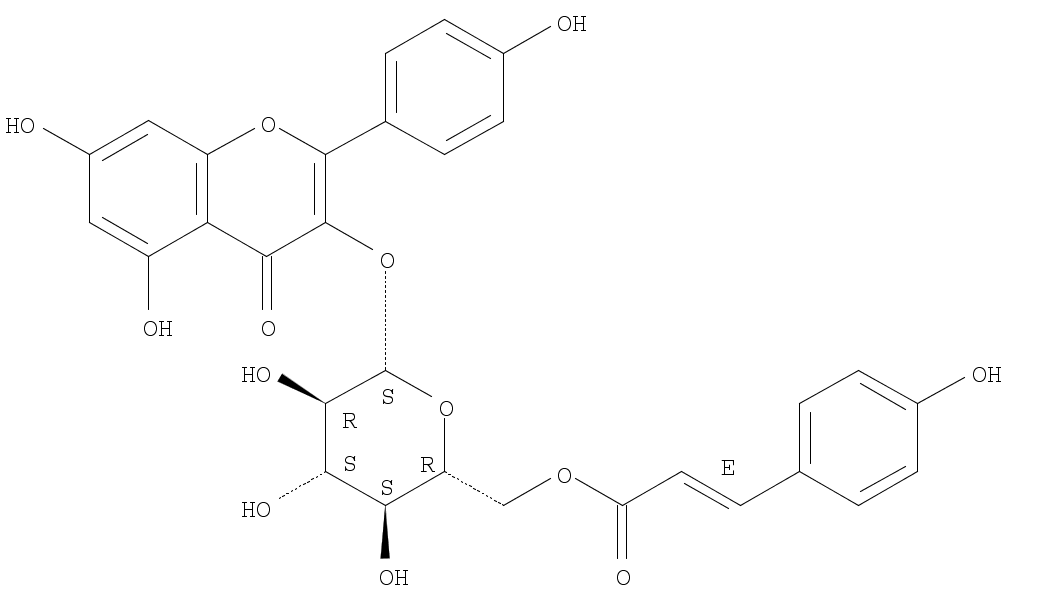 | >2000 | >2000 | >2000 | >2000 |
| Ursolic Acid [77-52-1] | 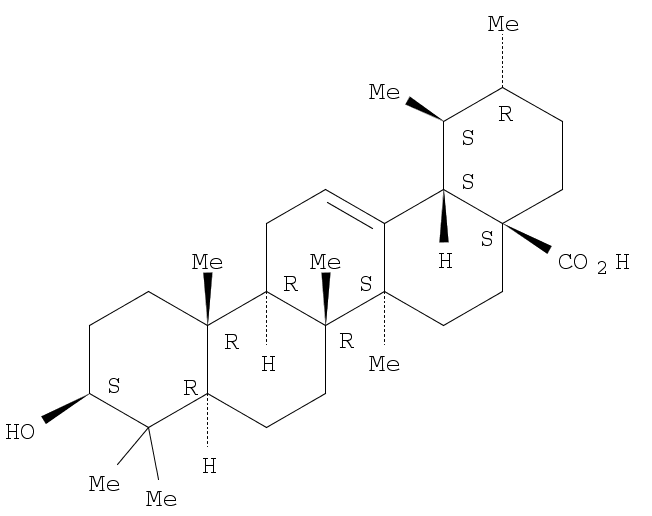 | 200 | 400 | 700 | 800 |
